# Whole Genome Sequencing Increases Molecular Diagnostic Yield Compared with Current Diagnostic Testing for Inherited Retinal Disease

**DOI:** 10.1016/j.ophtha.2016.01.009

**Published:** 2016-05

**Authors:** Jamie M. Ellingford, Stephanie Barton, Sanjeev Bhaskar, Simon G. Williams, Panagiotis I. Sergouniotis, James O'Sullivan, Janine A. Lamb, Rahat Perveen, Georgina Hall, William G. Newman, Paul N. Bishop, Stephen A. Roberts, Rick Leach, Rick Tearle, Stuart Bayliss, Simon C. Ramsden, Andrea H. Nemeth, Graeme C.M. Black

**Affiliations:** 1 Manchester Centre for Genomic Medicine, Saint Mary's Hospital, Central Manchester University Hospitals NHS Foundation Trust, Manchester Academic Health Science Centre, Manchester, United Kingdom; 2 Institute of Human Development, University of Manchester, Manchester, United Kingdom; 3 Manchester Royal Eye Hospital, Central Manchester University Hospitals NHS Foundation Trust, Manchester Academic Health Science Centre, Manchester, United Kingdom; 4 Institute of Population Health, University of Manchester, Manchester, United Kingdom; 5 Centre for Biostatistics, Institute of Population Health, University of Manchester, Manchester, United Kingdom; 6 Complete Genomics, Inc., Mountain View, California; 7 Nuffield Department of Clinical Neurosciences, John Radcliffe Hospital, University of Oxford, Oxford, United Kingdom

**Keywords:** CI, confidence interval, IRD, inherited retinal disease, MCGM, Manchester Centre for Genomic Medicine, NGS, next-generation sequencing, RP, retinitis pigmentosa, SNV, single nucleotide variant, WES, whole exome sequencing, WGS, whole genome sequencing

## Abstract

**Purpose:**

To compare the efficacy of whole genome sequencing (WGS) with targeted next-generation sequencing (NGS) in the diagnosis of inherited retinal disease (IRD).

**Design:**

Case series.

**Participants:**

A total of 562 patients diagnosed with IRD.

**Methods:**

We performed a direct comparative analysis of current molecular diagnostics with WGS. We retrospectively reviewed the findings from a diagnostic NGS DNA test for 562 patients with IRD. A subset of 46 of 562 patients (encompassing potential clinical outcomes of diagnostic analysis) also underwent WGS, and we compared mutation detection rates and molecular diagnostic yields. In addition, we compared the sensitivity and specificity of the 2 techniques to identify known single nucleotide variants (SNVs) using 6 control samples with publically available genotype data.

**Main Outcome Measures:**

Diagnostic yield of genomic testing.

**Results:**

Across known disease-causing genes, targeted NGS and WGS achieved similar levels of sensitivity and specificity for SNV detection. However, WGS also identified 14 clinically relevant genetic variants through WGS that had not been identified by NGS diagnostic testing for the 46 individuals with IRD. These variants included large deletions and variants in noncoding regions of the genome. Identification of these variants confirmed a molecular diagnosis of IRD for 11 of the 33 individuals referred for WGS who had not obtained a molecular diagnosis through targeted NGS testing. Weighted estimates, accounting for population structure, suggest that WGS methods could result in an overall 29% (95% confidence interval, 15–45) uplift in diagnostic yield.

**Conclusions:**

We show that WGS methods can detect disease-causing genetic variants missed by current NGS diagnostic methodologies for IRD and thereby demonstrate the clinical utility and additional value of WGS.

Defining, with precision, the molecular genetic basis of ophthalmic disorders has a profound influence on clinicians' ability to diagnose, counsel, treat, and manage their patients.^1^ Inherited retinal dystrophies (IRDs) are a diverse group of genetic disorders associated with visual impairment. They cause visual impairment in more than 2 million people worldwide and show extreme clinical and genetic heterogeneity.^2^ As such, the clinical application of next-generation sequencing (NGS), a relatively recent technological advance that allows fast and cost-effective detection of genetic variation through parallel sequencing of multiple copies of fragmented DNA,^3^ has transformed IRD discovery programs and genomic diagnostics, as well as for other Mendelian disorders.^4^ Next-generation DNA sequencing techniques permit the analysis of genetic variants across multiple areas of the genome in a single procedure. To date, the application of NGS in the clinic largely has been limited to targeted techniques such as custom gene panels^5^, ^6^, ^7^ and whole exome sequencing (WES),^8^, ^9^ which primarily restrict the analysis of genetic variation to protein-coding regions of the genome. However, these techniques are limited by the completeness of the genetic variation that can be detected, including reliable identification of large genomic deletions or duplications that encapsulate protein-coding regions and pathogenic variants in noncoding regions of genes. Such limitations are less evident from whole genome sequencing (WGS), an approach capable of detecting all types of variation across the complete human genome.^10^ Targeted NGS techniques are the most commonly used genomic diagnostic test for IRD, but decreasing costs and increasing data interpretability have made WGS a realistic prospect for diagnostic use in the clinic.^11^, ^12^, ^13^, ^14^ This is exemplified by recent large-scale WGS cohorts, for example, the “100,000 Genomes Project” in England.^15^ However, the comparative benefits of this technology, in respect to current diagnostic services, have yet to be truly delineated. The objective of this study is to identify the additional clinical advantages of WGS for individuals with IRD. We report the clinical findings from a retrospective review of 562 individuals referred for targeted NGS and a paired head-to-head comparison of targeted NGS and WGS for 46 unrelated individuals with clinical indications of IRD.

## Methods

### Sampling and Study Design

#### Targeted Next-Generation Sequencing Testing

The retrospective review included the first 562 individuals referred with IRD for clinically accredited targeted NGS. All individuals underwent clinical analysis of genetic variation within 105 genes known to underpin IRD (Table 1, available at www.aaojournal.org).^5^ The included individuals were not knowingly related and had been referred from worldwide ophthalmic institutions. We analyzed the information available in the molecular diagnostic report and genetic variant files, including analysis of variant consequences and pathogenicity, clinical outcome, and carrier status. Phenotype–genotype correlations were elucidated from the scientific literature, including those referenced at the Retinal Information Network.^16^ All analyses were conducted at the UK Manchester Centre for Genomic Medicine (MCGM).

#### Comparison of Whole Genome Sequencing and Targeted Next-Generation Sequencing Testing: Variant Detection Accuracy

To determine the ability of targeted NGS and WGS to detect single nucleotide variants (SNVs), we performed independent assessments of the *sensitivity* and *specificity* of the 2 techniques using control samples. We obtained control samples from the Coriell Institute for Medical Research Biorepositories in May 2013, which had been anonymized with unique catalog identifiers. Ethical permission was granted for the use of control samples to improve genomic diagnostic services, in line with the National Human Genome Research Institute Assurance Form for Biomaterials (B-031709, available at http://www.catalog.coriell.org). All control samples had publically available genotype data generated through the Illumina OMNI v2.5 microarray, a technique that identifies genotypes at approximately 2.5 million prespecified locations across the genome. We compared genotypes from the Illumina OMNI v2.5 microarray (available for each sample at ftp://ftp.sanger.ac.uk) with genotype calls from the targeted NGS and WGS pipelines. This was performed for 4 samples using targeted NGS through the Illumina HiSeq sequencing platform and 6 samples using WGS.

We calculated the *sensitivity* and *specificity* of targeted NGS and WGS to detect SNVs compared with the Illumina OMNI v2.5 microarray as described previously: *sensitivity*, the ability to detect SNVs identified by the Illumina OMNI v2.5 microarray, and *specificity*, the ability to identify homozygous reference sites identified by the Illumina OMNI v2.5 microarray.^17^ For the clinically analyzed region of 105 genes surveyed by both targeted NGS and WGS, we were able to compare genotypes with the Illumina OMNI v2.5 microarray for 2714 genotyped sites using targeted NGS (616 SNVs and 2098 homozygous reference sites) and 4166 genotyped sites using WGS (928 SNVs and 3238 homozygous reference sites). All discordant sites were assessed via Sanger sequencing,^18^ the “gold standard” genotyping technique.

#### Comparison of Whole Genome Sequencing and Targeted Next-Generation Sequencing Testing: Diagnostic Utility

**Figure 1:**
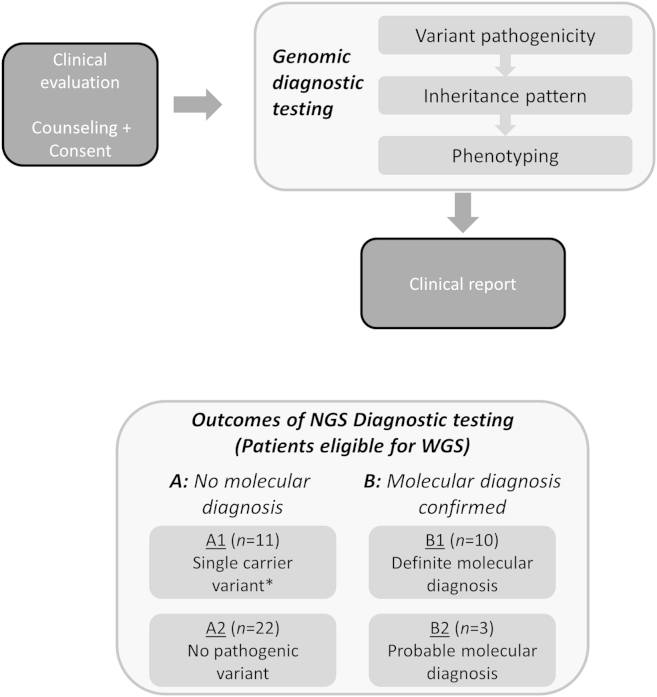
Data analysis and study design summary. Overview of targeted next-generation sequencing (NGS) diagnostic testing. *Single carrier variant defined as an individual with a pathogenic heterozygous variant found in a gene relevant to their clinical indication of inherited retinal disease (IRD) that is known to cause recessively inherited disease. WGS = whole genome sequencing.

The major motivation for this research was to assess whether WGS alters the number of individuals with a molecular diagnosis in comparison with currently delivered targeted genomic diagnostics. To address this question, we performed a paired head-to-head comparison between targeted NGS and WGS for 46 unrelated individuals with a clinical indication of IRD. All 46 individuals had been phenotypically assessed in a single institution. The cohort referred for WGS included the suite of possible clinical outcomes from targeted NGS testing (Fig 1). For each of the 46 individuals, we undertook consecutive genomic screening: first, targeted NGS, and second, targeted analysis of WGS data. For each individual, we assessed whether targeted NGS and WGS identified the same clinically relevant mutations and achieved the same clinical outcome.

Ethical permission for WGS was sought and granted for a single center, MCGM. Ethics Committee approval for this study was obtained through the REGARD study (Greater Manchester West Research Ethics Committee reference number: 11/NW/0421). Of the 562 individuals referred for targeted NGS testing, 126 were referred by clinicians at the MCGM, and 59 had appropriate consent for research genomics through the REGARD study. We excluded 13 of the 59 individuals on the basis of the quality and quantity of DNA available. The remaining 46 individuals were referred for WGS, which was performed using 10 μg of DNA that had been extracted from patient blood samples and stored during targeted NGS testing. All individuals referred for WGS were analyzed on a singleton basis and had received a clinical outcome from targeted NGS before January 2014. Targeted analysis was performed on WGS data within hierarchical lists of genes known to cause IRD (first for 105 genes, Table 1; second for 180 genes, Table 2, both available at www.aaojournal.org). The methodologies of this research adhered to the tenets of the Declaration of Helsinki.

### Sequencing and Variant Calling

#### Targeted Next-Generation Sequencing Testing

Targeted NGS diagnostic testing was performed for 562 patient DNA samples. The pipeline involved (i) enrichment of relevant genomic regions, (ii) multiplexed high-throughput DNA sequencing (NGS), (iii) clinical bioinformatics (demultiplexing, alignment to the *hg19* reference genome, variant calling and annotation), and (iv) clinical analysis and determination of pathogenicity of rare genomic variations.

The targeted capture region was defined as the protein-coding regions ±50 base pairs of specified transcripts for 105 genes (Table 1, available at www.aaojournal.org) and a specific noncoding region of the *CEP290* gene. Enrichment was performed using an Agilent SureSelect Custom Design target-enrichment kit (Agilent, Santa Clara, CA). Next-generation sequencing was performed using the manufacturer protocols for the ABI SOLiD 5500 platform (n = 255; Life Technologies Corporation, Carlsbad, CA) and the Illumina HiSeq 2000/2500 platform (n = 307; Illumina, Inc., San Diego, CA). Clinical bioinformatics was performed using a variety of open-source software, including Lifescope, CASAVA v.1.8.2., BWA-short v.0.6.2^19^ and GATK-lite v2.0.39.^20^ Annotations were performed using v68 of the Ensembl database.

#### Whole Genome Sequencing

Whole genome sequencing was performed on 52 DNA samples (6 controls, 46 patients) by Complete Genomics (Mountain View, CA) as described previously.^21^ Bioinformatics (alignment to the *hg19* reference genome, local de novo assembly, and variant calling) was performed using version 2.5 of the Complete Genomics pipeline.^22^ Variants were restricted to those in specified lists of genes (first for 105 genes, Table 1; second for 180 genes, Table 2, both available at www.aaojournal.org), and their presence was confirmed through an alternative method before they were clinically reported.

### Determining Clinical Outcomes

The clinical analysis of genomic variation requires the interpretation of pathogenicity, the assessment of genetic inheritance patterns, and detailed phenotypic analysis (Fig 1). These analyses determine the clinical outcome of diagnostic molecular testing, which may (i) define an unequivocal clinical diagnosis, (ii) confirm a likely clinical diagnosis, or (iii) exclude, challenge, or fail to confirm a clinical diagnosis. The detailed clinical algorithms used in each stage of the analysis procedure are included in the Appendix (available at www.aaojournal.org). Variants identified by WGS also were classified in accordance with recent clinical guidelines for genetic variant interpretation.^23^

### Projected Impact of Clinical Whole Genome Sequencing

To predict the potential impact of the WGS pipeline on current clinical practice, we derived a weighted estimate of the diagnostic yield based on the prevalence of clinical outcomes observed in the population of 562 patients referred for targeted NGS testing and the sampling proportions and diagnostic yield in the stratified sample of 46 patients referred for both targeted NGS testing and WGS. The 95% confidence interval (CI) was obtained by simulating draws from a binomial distribution for each of the estimated proportions and computing the 2.5 and 97.5 percentiles of the distribution of the resultant uplift estimates. R software was used to generate 10 000 simulations.

## Results

### Patient Characteristics

A total of 562 patients (male, n = 299; female, n = 263) were referred for targeted NGS testing from healthcare institutions in 12 different countries; the majority were referred from within the United Kingdom (78%; n = 436). Clinical indications of diagnoses derived from 1 of 10 distinct forms of IRD, the most common being retinitis pigmentosa (RP) (48%) (Table 3). All of the 46 patients who underwent WGS (male, n = 24; female, n = 22) were referred by clinicians in a single center. Of these, 34 had nonsyndromic IRD. Among the remaining 12 patients, additional clinical features suggesting a syndromic form of IRD included hearing loss, n = 9; hearing loss and renal failure, n = 1; hearing loss and gross motor delay, n = 1; and autism, learning difficulties and developmental delay, n = 1. Nonsyndromic RP also was the most common IRD subtype among the 46 patients referred for WGS (Table 3), accounting for 43% of the cohort.

### Coverage, Sensitivity, and Specificity of Whole Genome Sequencing and Targeted Next-Generation Sequencing

We defined coverage as the number of times a single nucleotide was sequenced by unique and independent NGS reads. We directly compared the coverage achieved by targeted NGS and WGS for the protein coding region of the 105 genes surveyed by both techniques and found that targeted NGS and WGS achieved ≥20× average coverage for 98.4% (n = 562) and 98.5% (n = 52), respectively. Whole genome sequencing achieved, on average, ≥20× coverage for 96.5% of the whole genome and 98.0% of the protein-coding region of the genome (n = 52) (Table 4, available at www.aaojournal.org). However, WGS generated inconsistent coverage at ≥20× across repetitive regions of the genome, such as *RPGRorf15* (mean = 58.0%, min/max = 39.0/78.1%, n = 52) (Table 4, available at www.aaojournal.org).

Compared with the Illumina OMNI v2.5 microarray genotyped sites within the protein-coding regions of 105 genes, WGS (surveyed SNVs, n = 928) and targeted NGS (surveyed SNVs, n = 616) achieved 100% sensitivity for SNV detection and 99.9% (surveyed sites, n = 3238) and 100% (surveyed sites, n = 2098) specificity for SNV detection, respectively. On the basis of our findings from Sanger sequencing discordant sites, an inherent error rate of 2.16% in the Illumina OMNI v2.5 microarray, we estimate that the WGS pipeline achieved 97.8% sensitivity and 99.3% specificity for SNV detection for the protein-coding and noncoding regions of 180 genes analyzed during the second tier of analysis (Tables 5–7, available at www.aaojournal.org). Likewise, we estimate that WGS achieved 97.7% sensitivity and 98.9% specificity for SNV detection for the whole genome (Table 8, available at www.aaojournal.org).

### Summary of Findings from Targeted Next-Generation Sequencing for 562 Individuals with Inherited Retinal Disease

**Figure 2:**
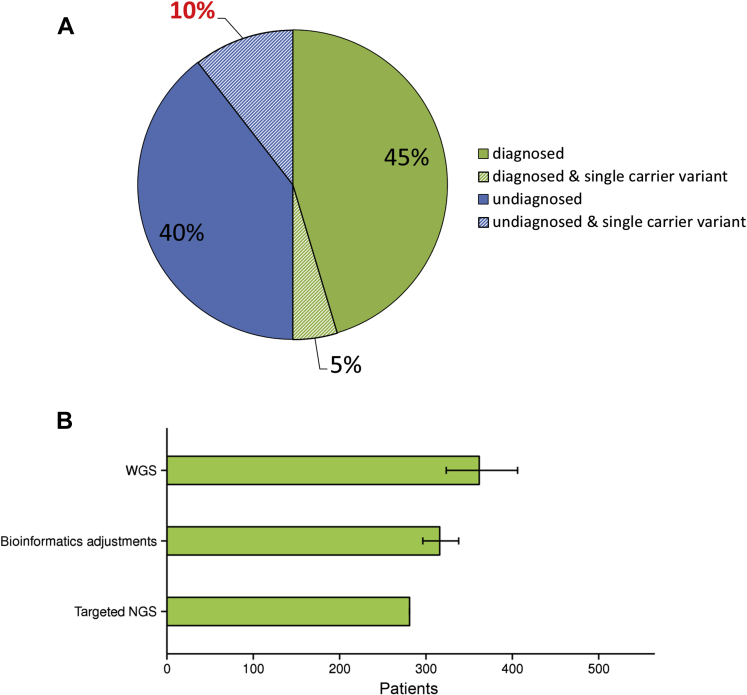
**A**, Clinical outcome of diagnostic next-generation sequencing (NGS) testing for 562 individuals. Diagnosed, individuals with a molecular diagnosis. Undiagnosed, individuals without a molecular diagnosis. Single carrier variant, individuals who are heterozygous for a “clearly pathogenic” or “likely pathogenic” variant in a gene associated with recessive retinal dystrophy; no second variant was identified. Other variants in additional genes may or may not be determined to cause the phenotype, this refers to diagnosed and single carrier variant and undiagnosed and single carrier variant, respectively. **B**, Projected impact of whole genome sequencing (WGS) on clinical molecular diagnostics. A weighted biased estimate with 95% confidence intervals (CIs) of the projected increase in molecular diagnostic yield: targeted NGS, number of individuals with a molecular diagnosis through targeted NGS diagnostics; bioinformatics adjustments, the number of individuals expected to receive a molecular diagnosis after alterations to the bioinformatics pipeline for targeted NGS diagnostics; WGS, the number of individuals expected to have a molecular diagnosis if WGS were to be applied to the 562 referred patients.

Targeted NGS testing identified 416 genetic variants likely or highly likely to account for disease presentation in 281 patients (50%) (Fig 2A), including 53 autosomal dominant cases, 1 X-linked dominant case, 13 X-linked recessive cases, and 214 autosomal recessive cases (91 homozygous cases and 123 compound heterozygous cases).

The Illumina HiSeq sequencing platform achieved ≥20× coverage across significantly more of the clinically analyzed region of 105 genes for referred individuals than the ABI SOLiD sequencing platform (HiSeq = 99.00±0.01, SOLiD = 97.63±0.06, *P* < 0.0001). However, there was no significant difference in the diagnostic success rate of the 2 sequencing platforms (HiSeq = 48%, SOLiD = 52%, *P* = 0.37).

Of the 562 referred patients, 28% (n = 158) were reported to carry heterozygous variants that would be expected to cause disease if present in a compound heterozygous or homozygous state (i.e., carrier status of recessive trait). For 61 of these patients (39%), the identification of clinically relevant variants in other genes achieved an alternative molecular diagnosis (dominant cases, n = 15; recessive cases, n = 46). The remaining 97 patients (61%) did not receive a molecular diagnosis, 59 of whom had heterozygous variants in genes known to be a cause of their specific disorder (Fig 2A; Table 3 shows a list of all IRD subtypes).

### Head-to-Head Comparison of Whole Genome Sequencing and Targeted Next-Generation Sequencing for 46 Individuals with Inherited Retinal Disease

Forty-six of the 562 patients who had been analyzed by targeted NGS were subsequently analyzed using a WGS pipeline, including 13 individuals with a confirmed or provisionally confirmed molecular diagnosis and 33 individuals without a molecular diagnosis. Whole genome sequencing found more molecular diagnoses than were initially identified by targeted NGS techniques (targeted NGS = 13, WGS = 24). The increase in molecular diagnoses was underpinned by the detection of additional clinically relevant variants accounting for or likely accounting for disease presentation in 11 of the individuals without a molecular diagnosis from targeted NGS (Table 9).

For 13 individuals with a molecular diagnosis from targeted NGS, we assessed whether WGS identified the same molecular diagnosis. We identified 10 “clearly pathogenic” and 9 “likely pathogenic” variants (missense, n = 9; nonsense, n = 6; frameshift, n = 4) (Table 10, available at www.aaojournal.org) accounting for 10 confirmed molecular diagnoses and 3 provisional molecular diagnoses, all of which had been identified by targeted NGS. Through WGS, we also identified 2 “likely pathogenic” heterozygous deletions that had not been identified through targeted NGS testing (Table 9) (*067429* and *12008422*). A heterozygous deletion in *USH2A* was identified *in trans* to a missense variant that had been determined as homozygous and “likely pathogenic” through targeted NGS. This finding refined the molecular diagnosis for the referred individual (Table 10, available at www.aaojournal.org) (*12008422*). A heterozygous deletion in *RPGRIP1* was identified in an individual referred with sporadic RP. This finding was not thought to contribute to the molecular diagnosis for the referred individual (Table 10, available at www.aaojournal.org) (*067429*) because “clearly pathogenic” and “likely pathogenic” variants were found in another gene, *EYS*, and mutations in *RPGRIP1* are expected to cause recessively inherited disease.^24^ Both identified *RPGRIP1* and *USH2A* heterozygous deletions have counseling implications for the referred individuals.

For 33 individuals without a molecular diagnosis from targeted NGS, we found that the WGS pipeline identified 9 “likely pathogenic” variants and 3 “clearly pathogenic” variants that had not been identified by targeted NGS (Table 9). These included 3 large deletions encapsulating protein-coding regions, 5 variants in noncoding regions of genes, 3 complex insertion and deletion events, and 1 variant in an additional gene implicated in the onset of IRD that is not included in the 105 gene–targeted NGS test. The variants underpinning IRD in each of the 46 patients with WGS data have been submitted to the ClinVar database (Accession numbers: SCV00259071-SCV000259109). We outline the patient benefit provided by WGS for an exemplar individual in the included Case Study.

If the WGS pipeline is applied to all 562 patients referred for targeted NGS testing, and assuming the same rate of mutation discovery, we calculate that the WGS pipeline could provide a 29% (95% CI, 15–45) uplift in diagnostic yield compared with current targeted NGS services (Fig 2B). However, much of this uplift also could be achieved by straightforward modifications to the targeted NGS bioinformatics pipeline (12%, 95% CI, 5–20) (Fig 2B), including the use of additional variant calling software (e.g., Pindel^25^) and corrections to the regions of the genome that are clinically analyzed (e.g., extension of the analysis to known pathogenic variants in noncoding regions of the genome) (Table 11, available at www.aaojournal.org).

## Discussion

Our ability to interrogate the human genome in a clinical setting has transformed rapidly over the past decade. The present day genomic diagnostic laboratory now hosts an arsenal of high-throughput DNA surveillance techniques. These include DNA sequencing specifically targeted to areas of the genome already known to harbor disease-causing variation, for example, the targeted sequencing (described in this article) of protein-coding regions for 105 genes implicated in the onset of IRD. Whole genome sequencing now offers an opportunity to analyze variation across the whole human genome, and because of reductions in cost and increases in data interpretability offers a potential further step-change in the clinical evaluation of genetic variation for rare (Mendelian) disease, including the highly heterogeneous group of IRD. Whole genome sequencing has the potential to become an additional tool for genome diagnostic laboratories. However, the additional advantages of WGS over current genomic diagnostic technologies (targeted NGS) for ophthalmic disorders are not yet fully quantified, because formal comparative analyses of the 2 techniques have not been undertaken.

**Figure 3:**
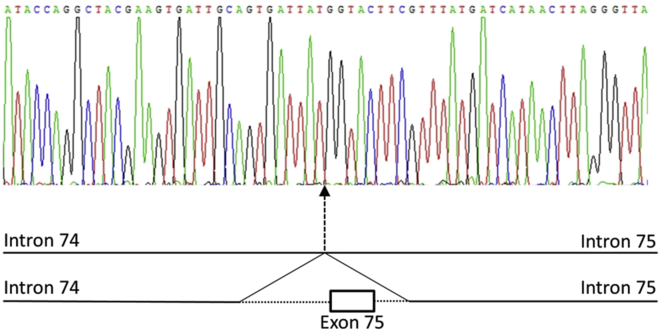
Sanger sequencing chromatogram showing the breakpoints of a heterozygous deletion removing a single exon from the reading frame of the *GPR98* gene.

We surveyed 562 patients referred for targeted NGS with clinical indications of IRD (Table 3) and established a molecular diagnosis in 50% of cases, a rate consistent with other published figures.^6^, ^7^, ^8^, ^26^ Our findings confirm the power of targeted NGS approaches to provide a diagnosis for individuals with IRD. Although the transition from the ABI SOLiD sequencing platform to the Illumina HiSeq sequencing platform did not increase diagnostic yield for referred patients, it has increased the clarity of diagnostic reporting because it achieved more uniform sequencing coverage and a greater percentage of DNA nucleotides sequenced at specified diagnostic thresholds. Of note, among those with a positive diagnosis from targeted NGS, 5% carry single heterozygous pathogenic variants in genes known to cause recessive IRD and identified in genes relevant to their specific phenotype. Such carrier variants are at a higher frequency in individuals without a molecular diagnosis (Fig 2A), leading to uncertainty in diagnosis. This highlights potential limitations of targeted NGS technologies to define the true diversity of genetic variation underpinning IRD. To assess these limitations, 46 of the 562 patients underwent WGS to compare the mutation detection rates and molecular diagnostic yield with those achieved by targeted NGS. We clearly demonstrate that WGS pipelines have a greater power than current targeted NGS strategies for detecting a wider spectrum of genetic variants (Table 9), including large structural variation, noncoding mutations excluded from targeted NGS analysis regions, and mutations in genes excluded from targeted capture. The deletions identified by the WGS pipeline ranged from <1.7 Kb to >520 Kb in size, and the identification of breakpoints in noncoding regions of the genome permitted subsequent cost-effective clinical validation (Fig 3). Therefore, this work highlights the benefit of a single diagnostic technique for IRD that can accurately identify disease-causing small variation and large structural variation encompassing protein-coding regions of genes (see Case Study for illustrative example). Traditionally, this has been possible only through a combination of 2 or more diagnostic modalities because software to detect large structural variation from targeted NGS data (e.g., ExomeDepth^27^) remains unvalidated in a clinical diagnostic context.

From a diagnostic viewpoint, we also assessed the sensitivity and specificity of targeted NGS and WGS to detect genetic variation of single nucleotides (SNVs), using 6 control samples. We compared genotypes generated through targeted NGS and WGS with those publically available from the Illumina OMNI v2.5 microarray (available at ftp://ftp.sanger.ac.uk) and found that the WGS pipeline achieved similar sensitivity and specificity levels for SNV detection to targeted NGS and demonstrated better sensitivity and specificity levels for SNV detection than those reported for WES pipelines.^17^ This is consistent with data suggesting that WGS is a better tool for genetic variant detection in protein-coding regions than WES.^10^, ^28^ It is of note that both techniques shared limitations, for example, both targeted NGS and WGS were unable to reproducibly survey the repetitive and AG rich protein-coding region of *RPGR* (orf15) (Table 4, available at www.aaojournal.org), which accounts for 75% of X-linked RP.^29^

The data underline the clinical utility of NGS modalities for diagnosing IRD and confirm the considerable utility of targeted NGS as a diagnostic tool for IRD. Furthermore, the analysis of WGS for a small cohort of individuals has improved the routine diagnostic services provided for individuals referred with IRD. The costs and data burdens associated with WGS remain substantial; therefore, the continual translation of findings from WGS into currently delivered molecular diagnostics will, in the short term, provide the best route for developing more sensitive diagnostic tests for IRD. However, in assessing the limitations of current diagnostic modalities, and in looking to the future, we demonstrate the additional benefits of clinical WGS in surveying genomic variation (Fig 2B). As the cost and data burdens associated with WGS decrease sufficiently, whole genome-based sequencing pipelines are likely to become routinely used in the diagnosis of IRD.

### Case Study: Usher Syndrome

A female presented with RP and congenital deafness, suggesting a clinical diagnosis of Usher syndrome. There was no family history.

Targeted NGS testing identified no molecular cause. The patient carried the following:

i) a “likely pathogenic” heterozygous variant in *IDH3B*, c.184G>T, p.(Glu62*).

Mutations in *IDH3B* have been associated with autosomal recessive nonsyndromic RP.^30^ Therefore, this finding was concluded not to contribute to the molecular diagnosis of IRD for this individual.

Analysis of WGS data also identified the following:

ii) a “likely pathogenic” heterozygous *GPR98* noncoding variant, c.1239-8C>G
iii) a “likely pathogenic” heterozygous deletion, c.16079-1456_ c.16196+155del p.(Ser5361Profs*25), removing a protein-coding region (exon) of *GPR98* (Fig 3) and expected to cause premature termination of protein translation.

Homozygous and compound heterozygous mutations in GPR98 cause Usher syndrome.^2^ These findings were concluded to confirm a diagnosis of autosomal recessive Usher syndrome.

## Figures and Tables

**Table 3:** Clinical Indications of Patients Referred for Targeted Next-Generation Sequencing and Whole Genome Sequencing

Clinical Diagnosis	No. of Cases Referred for Targeted NGS	No. of Cases Referred for WGS
RP or rod-cone dystrophy	268	20
Leber congenital amaurosis or early onset rod-cone dystrophy	78	4
Other (indication not included in this list, or not defined)	43	5
Stargardt disease or macular dystrophy	49	5
Usher syndrome	41	8
Cone-rod dystrophy	39	3
Achromatopsia or cone dystrophy	27	1
Syndromic ciliopathies	8	-
Familial exudative vitreoretinopathy	5	-
Choroideremia	4	-

NGS = next-generation sequencing; RP = retinitis pigmentosa; WGS = whole genome sequencing.

**Table 9:** Clinically Relevant Variants Identified by Whole Genome Sequencing That Were Not Detected by Targeted Next-Generation Sequencing Testing

Patient ID	Gene	Zygosity	cDNA	Protein	Genomic Coordinates (*hg19)*
Large Deletions					
12002355	*PCDH15*	Heterozygous	c.-189197_c.610-5166del	Removes start codon	Chr10:56,094,632-56,613,219
065240	*MERTK*	Homozygous	c.-8163_c.1145-1213del	Removes start codon	Chr2:112,648,150-112,739,206
11012351	*GPR98*	Heterozygous	c.16079-1455_c.16196+155del	p.(Ser5361Profs∗25)	Chr5:90,109,981-90,111,708
12008422	*USH2A*	Heterozygous	c.6326-3582_6658-1028del	p.(Asp2109Glyfs∗11)	Chr1:216,167,537-216,177,486
067429	*RPGRIP1*	Heterozygous	c.2710+485_3238+810del	p.(Gly904_Asn1079del)	Chr14:21,794,817-21,799,356
Intronic Variants					
09006916	*ABCA4*	Heterozygous	c.5461-10T>C	n/a	Chr1:94,476,951
12007903	*ABCA4*	Heterozygous	c.5461-10T>C	n/a	Chr1:94,476,951
11012351	*GPR98*	Heterozygous	c.1239-8C>G	n/a	Chr5:89,924,371
Insertions-Deletions					
11001193	*PDE6B*	Heterozygous	c.1923_1969delinsTCTGGG	p.(Asn643Glyfs∗29)	Chr4:654,564-657,607
11013807	*USH2A*	Heterozygous	c.5614delinsTTAACTTGGCAT	p.(Ala1872Metfs∗4)	Chr1:216,246,601
12003183	*CRX*	Heterozygous	c.648delC	p.(Ser216Argfs∗3)	Chr19:48342972
Missed by Informatics Errors					
065238	*ABCA4*	Heterozygous	c.5714+5G>A	n/a	Chr1:94,476,351
13012708	*ABCA4*	Heterozygous	c.5714+5G>A	n/a	Chr1:94,476,351
Variants in Additional 75 Genes					
11012959	*TRPM1*	Homozygous	c.707T>C	p.(Leu236Pro)	Chr15:31,358,296

n/a = not available.

∗ Indicates a premature termination during protein translation. All variant confirmations are available online in Figures 4 to 17, available at www.aaojournal.org.
