# Supplementary material for: Whole Genome Sequencing Increases Molecular Diagnostic Yield Compared with Current Diagnostic Testing for Inherited Retinal Disease
Source: Ophthalmology. 2016 May;123(5):1143–50. doi: 10.1016/j.ophtha.2016.01.009 (PMC4845717; doi:10.1016/j.ophtha.2016.01.009)
Supplement: Table 1 [file mmc1.pdf]

Table 1. Specified transcripts for 105 gene symbols analysed by targeted NGS diagnostic testing.

HGNC symbol	RefSeq Transcript ID	HGNC symbol	RefSeq Transcript ID	HGNC symbol	RefSeq Transcript ID
ABCA4	NM_000350.2	*PRPF31*	NM_015629.3	*IMPG2*	NM_016247.3
ADAM9	NM_003816.2	*PRPF6*	NM_012469.3	*KCNV2*	NM_133497.3
AIPL1	NM_014336.3	*PRPF8*	NM_006445.3	*KLHL7*	NM_001031710.2
ARL6	NM_032146.3	*PRPH2*	NM_000322.4	*IMPG2*	NM_016247.3
BBS1	NM_024649.4	*RAX2*	NM_032753.3	*KCNV2*	NM_133497.3
BBS10	NM_024685.3	*RBP3*	NM_002900.2	*KLHL7*	NM_001031710.2
BBS12	NM_001178007.1	*RD3*	NM_183059.2	*LCA5*	NM_181714.3
BBS2	NM_031885.3	*RDH12*	NM_152443.2	*LRAT*	NM_004744.3
BBS4	NM_033028.3	*RDH5*	NM_001199771.1	*LRP5*	NM_002335.2
BBS5	NM_152384.2	*RGR*	NM_002921.3	*MERTK*	NM_006343.2
BBS7	NM_176824.2	*RGS9*	NM_001165933.1	*MKKS*	NM_018848.2
BBS7	NM_018190.3	*RGS9*	NM_003835.3	*MKS1*	NM_001165927.1
BBS9	NM_198428.2	*RHO*	NM_000539.3	*MKS1*	NM_017777.3
BEST1	NM_004183.3	*RIMS1*	NM_014989.4	*MYO7A*	NM_000260.3
C1QTNF5	NM_015645.3	*RIMS1*	NM_001168407.1	*NDP*	NM_000266.3
C2orf71	NM_001029883.1	*CRB1*	NM_201253.2	*RIMS1*	NM_001168410.1
CA4	NM_000717.3	*CRX*	NM_000554.4	*RLBP1*	NM_000326.4
CACNA2D4	NM_172364.4	*DFNB31*	NM_015404.3	*ROM1*	NM_000327.3
CDH23	NM_022124.5	*DHDDS*	NM_024887.2	*RP1*	NM_006269.1
CDHR1	NM_001171971.1	*EFEMP1*	NM_001039348.2	*RP1L1***	NM_178857.5
CDHR1	NM_033100.2	*ELOVL4*	NM_022726.3	*RP2*	NM_006915.2
*CEP290**	NM_025114.3	*EYS*	NM_001142800.1	*RP9*	NM_203288.1
CERKL	NM_001030311.2	*FAM161A*	NM_001201543.1	*RPE65*	NM_000329.2
CHM	NM_000390.2	*FSCN2*	NM_001077182.2	*RPGR****	NM_001034853.1
CLRN1	NM_052995.2	*FZD4*	NM_012193.3	*RPGRIP1*	NM_020366.3
CLRN1	NM_001195794.1	*GNAT2*	NM_005272.3	*RS1*	NM_000330.3
CNGA1	NM_001142564.1	*GPR98*	NM_032119.3	*SAG*	NM_000541.4
CNGA3	NM_001298.2	*GUCA1A*	NM_000409.3	*SEMA4A*	NM_022367.3
CNGB1	NM_001297.4	*GUCA1B*	NM_002098.5	*SNRNP200*	NM_014014.4
CNGB3	NM_019098.4	*GUCY2D*	NM_000180.3	*SPATA7*	NM_018418.4
NR2E3	NM_014249.2	*IDH3B*	NM_006899.2	*TEAD1*	NM_021961.5
NRL	NM_006177.3	*IDH3B*	NM_174855.1	*TIMP3*	NM_000362.4
OTX2	NM_021728.2	*IMPDH1*	NM_000883.3	*TOPORS*	NM_005802.4
PCDH15	NM_001142763.1	*IMPG2*	NM_016247.3	*TRIM32*	NM_012210.3
PCDH15	NM_001142769.1	*KCNV2*	NM_133497.3	*TTC8*	NM_144596.2
PCDH15	NM_001142771.1	*KLHL7*	NM_001031710.2	*TULP1*	NM_003322.3
PCDH15	NM_001142770.1	*LCA5*	NM_181714.3	*UNC119*	NM_005148.3
PDE6A	NM_000440.2	*LRAT*	NM_004744.3	*UNC119*	NM_054035.2
PDE6B	NM_000283.3	*LRP5*	NM_002335.2	*USH1C*	NM_005709.3
PDE6C	NM_006204.3	*MERTK*	NM_006343.2	*USH1C*	NM_153676.3
PDE6G	NM_002602.3	*MKKS*	NM_018848.2	*USH1G*	NM_173477.2
PITPNM3	NM_031220.3	*MKS1*	NM_001165927.1	*USH2A*	NM_206933.2
PRCD	NM_001077620.2	*MKS1*	NM_017777.3	*ZNF513*	NM_144631.5
PROM1	NM_006017.2	*MYO7A*	NM_000260.3		
PRPF3	NM_004698.2	*NDP*	NM_000266.3		

* Testing of the common intron 26 mutation c.2991+1655A>G in CEP290 is included in this analysis
** Analysis of the coding region of exon 4 of the RP1L1 gene is not included
*** Analysis of the coding region of the final exon (*orf15*) of *RPGR* it is not included *HGNC*, gene symbols approved by the HUGO Gene Nomenclature Committee[1]

1. Gray KA, Yates B, Seal RL, Wright MW, Bruford EA. Genenames.org: the HGNC resources in 2015. Nucleic Acids Res 2015;43:D1079-85.
