# Supplementary material for: Whole Genome Sequencing Increases Molecular Diagnostic Yield Compared with Current Diagnostic Testing for Inherited Retinal Disease
Source: Ophthalmology. 2016 May;123(5):1143–50. doi: 10.1016/j.ophtha.2016.01.009 (PMC4845717; doi:10.1016/j.ophtha.2016.01.009)
Supplement: Table 2 [file mmc2.pdf]

Table 2. Gene symbols approved by the HUGO Gene Nomenclature Committee[1] for the 180 genes previously associated with inherited retinal disease utilized during the analysis of whole genome sequencing data.

Gene Symbol	MIM ID	Gene Symbol	MIM ID	Gene Symbol	MIM ID
ABCA4	601691	*CNGA1*	123825	*IQCB1*	609237
ABHD12	613599	*CNGA3*	600053	*ITM2B*	603904
ACBD5	23338*	*CNGB1*	600724	*KCNJ13*	603208
ADAM9	602713	*CNGB3*	605080	*KCNV2*	607604
ADAMTS18	607512	*CNNM4*	607805	*KIAA1549*	613344
AHI1	608894	*CRB1*	604210	*KIF11*	148760
AIPL1	604392	*CRX*	602225	*KLHL7*	611119
ALMS1	606844	*CSPP1*	611654	*LCA5*	611408
ARL2BP	615407	*CYP4V2*	608614	*LRAT*	604863
ARL6	608845	*DFNB31*	607084	*LRIT3*	615004
BBIP1	615995	*DHDDS*	608172	*LRP5*	603506
BBS1	209901	*DTHD1*	37261*	*LZTFL1*	606568
BBS10	610148	*EFEMP1*	601548	*MAK*	154235
BBS12	610683	*ELOVL4*	605512	*MERTK*	604705
BBS2	606151	*EMC1*	28957*	*MFRP*	606227
BBS4	600374	*EYS*	612424	*MKKS*	604896
BBS5	603650	*FAM161A*	613596	*MKS1*	609883
BBS7	607590	*FLVCR1*	609144	*MVK*	251170
BBS9	615986	*FSCN2*	607643	*MYO7A*	276903
BEST1	607854	*FZD4*	604579	*NDP*	300658
C1QTNF5	608752	*GNAT1*	139330	*NEK2*	604043
C21orf2	603191	*GNAT2*	139340	*NMNAT1*	608700
C2orf71	613425	*GNPTG*	607838	*NPHP1*	607100
C8orf37	614477	*GPR125*	612303	*NPHP3*	608002
CA4	114760	*GPR179*	614515	*NPHP4*	607215
CABP4	608965	*GPR98*	602851	*NR2E3*	604485
CACNA1F	300110	*GRK1*	180381	*NRL*	162080
CACNA2D4	608171	*GRM6*	604096	*NYX*	300278
CAPN5	602537	*GUCA1A*	600364	*OAT*	613349
CC2D2A	612013	*GUCA1B*	602275	*OFD1*	300170
CDH23	605516	*GUCY2D*	600179	*OTX2*	600037
CDH3	114021	*HARS*	142810	*PANK2*	606157
CDHR1	609502	*HMX1*	142992	*PCDH15*	605514
CEP164	614848	*IDH3B*	604526	*PCYT1A*	123695
CEP290	610142	*IFT140*	614620	*PDE6A*	180071
CERKL	608381	*IMPDH1*	146690	*PDE6B*	180072
CHM	300390	*IMPG1*	602870	*PDE6C*	600827
CIB2	605564	*IMPG2*	607056	*PDE6G*	180073

Gene Symbol	MIM ID	Gene Symbol	MIM ID	Gene Symbol	MIM ID
CLN3	607042	*INPP5E*	613037	*PEX1*	602136
CLRN1	606397	*INVS*	243305	*PEX2*	170993
PEX7	601757	*RGS9*	604067	*TEAD1*	189967
PHYH	602026	*RHO*	180380	*TIMP3*	188826
PITPNM3	608921	*RIMS1*	606629	*TMEM237*	614423
PLA2G5	601192	*RLBP1*	180090	*TOPORS*	609507
PRCD	610598	*ROM1*	180721	*TRIM32*	602290
PROM1	604365	*RP1*	603937	*TRPM1*	603576
PRPF3	607301	*RP1L1*	608581	*TSPAN12*	613138
PRPF31	606419	*RP2*	300757	*TTC8*	608132
PRPF4	607795	*RP9*	607331	*TUB*	601197
PRPF6	613979	*RPE65*	180069	*TULP1*	602280
PRPF8	607300	*RPGR*	312610	*UNC119*	604011
PRPH2	179605	*RPGRIP1*	605446	*USH1C*	605242
RAB28	612994	*RPGRIP1L*	610937	*USH1G*	607696
RAX2	610362	*RS1*	300839	*USH2A*	608400
RBP3	180290	*SAG*	181031	*VCAN*	118661
RBP4	180250	*SDCCAG8*	613524	*VPS13B*	607817
RD3	180040	*SEMA4A*	607292	*WDPCP*	613580
RDH12	608830	*SLC24A1*	603617	*WDR19*	608151
RDH5	601617	*SNRNP200*	601664	*ZNF423*	604557
RGR	600342	*SPATA7*	609868	*ZNF513*	613598

*Numerical identifications approved by the HUGO Gene Nomenclature Committee are given when MIM numerical identifiers are unavailable.

1. Gray KA, Yates B, Seal RL, Wright MW, Bruford EA. Genenames.org: the HGNC resources in 2015. Nucleic Acids Res 2015;43:D1079-85.
