# Supplementary material for: Whole Genome Sequencing Increases Molecular Diagnostic Yield Compared with Current Diagnostic Testing for Inherited Retinal Disease
Source: Ophthalmology. 2016 May;123(5):1143–50. doi: 10.1016/j.ophtha.2016.01.009 (PMC4845717; doi:10.1016/j.ophtha.2016.01.009)
Supplement: Table 4 [file mmc3.pdf]

Table 4. Coverage statistics achieved for 52 samples (6 control, 46 patients) referred for whole genome sequencing. Statistics correspond to (i) *genome*, the whole genomic region, (ii) *exome*, the region classified as protein coding in the *hg19* reference genome by Complete Genomics, (iii) *105-genes*, the targeted analysis region of 105 genes utilized during conventional NGS testing (Table 1, available at http://www.aaojournal.org/), (iv) *180-genes*, the exonic and intronic region of 180 genes utilized during the analysis of whole genome sequencing data (Table 2, available at http://www.aaojournal.org/), and (v) *RPGRorf15*, the region classified as the final exon (*orf15*) of the *RPGR* gene which is traditionally hard to sequence through next generation DNA sequencing techniques. All numerical values represent the percentage of the specified region that is covered at the specified number of sequencing reads.

	**Genome**		**Exome**		**105-genes**		**180-genes**		***RPGRorf15***	
**Coverage**	**average**	**(min-max)**	**average**	**(min-max)**	**average**	**(min-max)**	**average**	**(min-max)**	**average**	**(min-max)**
**≥10x**	99.1	98.8-99.2	99.6	99.2-99.7	99.9	99.4-99.9	99.2	98.7-99.4	78.8	63.2-93.6
**≥20x**	96.5	94.8-97.4	98.0	96.3-98.8	98.5	97.2-99.3	96.7	94.6-97.5	58.0	39.0-78.1
**≥30x**	89.5	84.8-92.5	92.8	87.9-95.5	93.3	88.8-96.0	89.6	83.3-92.3	37.6	9.8-65.7
**≥40x**	76.1	67.0-81.8	80.3	70.5-86.4	80.2	70.2-85.7	74.8	62.5-80.5	23.0	0.2-57.0
