# Supplementary material for: Whole Genome Sequencing Increases Molecular Diagnostic Yield Compared with Current Diagnostic Testing for Inherited Retinal Disease
Source: Ophthalmology. 2016 May;123(5):1143–50. doi: 10.1016/j.ophtha.2016.01.009 (PMC4845717; doi:10.1016/j.ophtha.2016.01.009)
Supplement: Table 5 [file mmc4.pdf]

Table 5. Sensitivity of the WGS pipeline for six control samples after confirmation of discordant sites through Sanger sequencing. Regions of interest include the clinically analysed region of 105 genes (Table 1, available at http://www.aaojournal.org/) and the hypothesized enrichment region (+/- 50 intronic base pairs of the coding region) for 180 genes (Table 2, available at http://www.aaojournal.org/). *HG* and *NA* refer to the Coriell Institute for Medical Research (https://www.coriell.org/) identifiers for the DNA samples used in the analysis.

Coriell ID	# sites surveyed	# observed no-calls	# observed discordances	# observed concordances	True positives	Real false negatives	Sensitivity
**105-genes**							
HG01970	134	0	1	133	134	0	1.0
NA18533	136	0	3	133	136	0	1.0
NA18907	180	0	3	177	180	0	1.0
NA19005	128	0	4	124	128	0	1.0
NA19194	174	0	4	170	174	0	1.0
NA19258	176	0	3	173	176	0	1.0
							**1.0**
**180-genes**							
HG01970	296	1	4	291	295	1	0.997
NA18533	312	1	7	304	311	1	0.997
NA18907	385	2	8	375	383	2	0.995
NA19005	290	0	8	282	290	0	1.0
NA19194	378	2	10	366	376	2	0.995
NA19258	374	2	8	364	372	2	0.995
							**0.996**

True positives, (i) WGS genotype matches Illumina OMNI microarray genotype (*# observed concordances*), or (ii) WGS genotype does not match Illumina OMNI microarray genotype (*# observed discordances*) but Sanger sequencing has proven WGS genotype is correct (see Table 6). *Real false negatives*, (i) WGS genotype has not been identified (*# observed no-calls*), or (ii) WGS genotype does not match Illumina OMNI microarray genotype (*# observed discordances*) and Sanger sequencing has proven WGS genotype is incorrect.
