# Supplementary material for: Whole Genome Sequencing Increases Molecular Diagnostic Yield Compared with Current Diagnostic Testing for Inherited Retinal Disease
Source: Ophthalmology. 2016 May;123(5):1143–50. doi: 10.1016/j.ophtha.2016.01.009 (PMC4845717; doi:10.1016/j.ophtha.2016.01.009)
Supplement: Table 6 [file mmc5.pdf]

Table 6. Proven errors in the Illumina OMNI v2.5 microarray at discordant sites detected between the Illumina OMNI v2.5 microarray and the WGS pipeline for six control samples. *HG* and *NA* refer to the Coriell Institute for Medical Research (https://www.coriell.org/) identifiers for the DNA samples used in the analysis.

Genomic co-ordinate (*hg19*)	Gene	Annotation	Reference	OMNI microarray		WGS pipeline		Sanger sequencing	
				**Allele 1**	**Allele 2**	**Allele 1**	**Allele 2**	**Allele 1**	**Allele 2**
HG01970									
chr1:94517166	*ABCA4*	c.2653+23	**G**	C	C	G	G	G	G
chr3:150690476	*CLRN1*	c.20	**T**	A	A	T	T	T	T
chr4:15982166	*PROM1*	c.2374-6	**A**	G	G	A	A	A	A
chr11:76895772	*MYO7A*	c.3503+12	**G**	A	A	del22	del22	del22	del22
NA18533									
chr1:94517166	*ABCA4*	c.2653+23	**G**	C	C	G	G	G	G
chr2:29287926	*C2orf71*	c.3676	**C**	C	T	C	C	C	C
chr3:150690476	*CLRN1*	c.20	**T**	A	A	T	T	T	T
chr4:15982166	*PROM1*	c.2374-6	**A**	A	G	A	A	A	A
chr11:61725599	*BEST1*	c.715-19	**A**	C	C	A	A	A	A
chr11:61725615	*BEST1*	c.715-3	**C**	T	T	C	C	C	C
chr16:53672338	*RPGRIP1L*	c.2959-15	**A**	A	G	A	A	A	A
NA18907									
chr1:94517166	*ABCA4*	c.2653+23	**G**	C	C	G	G	G	G
chr2:29287926	*C2orf71*	c.3676	**C**	C	T	C	C	C	C
chr3:150690476	*CLRN1*	c.20	**T**	A	A	T	T	T	T
chr4:15982166	*PROM1*	c.2374-6	**A**	A	G	A	A	A	A
chr10:95405667	*PDE6C*	c.1848-50	**A**	G	G	del2	del2	del2	del2
chr11:61725599	*BEST1*	c.715-19	**A**	C	C	A	A	A	A
chr11:61725615	*BEST1*	c.715-3	**C**	T	T	C	C	C	C
chr11:76895772	*MYO7A*	c.3503+12	**G**	G	A	G	del22	G	del22

Genomic co-ordinate (*hg19*)	Gene	Annotation	Reference	OMNI microarray		WGS pipeline		Sanger sequencing	
NA19005									
chr1:94517166	*ABCA4*	c.2653+23	**G**	C	C	G	G	G	G
chr2:29287926	*C2orf71*	c.3676	**C**	T	T	C	C	C	C
chr3:150690476	*CLRN1*	c.20	**T**	A	A	T	T	T	T
chr4:15982166	*PROM1*	c.2374-6	**A**	A	G	A	A	A	A
chr10:73553177	*CDH23*	c.6492	**C**	C	T	C	C	C	C
chr11:61725599	*BEST1*	c.715-19	**A**	C	C	A	A	A	A
chr11:61725615	*BEST1*	c.715-3	**C**	T	T	C	C	C	C
chr11:76895772	*MYO7A*	c.3503+12	**G**	G	A	G	del22	G	del22
NA19194									
chr1:94517166	*ABCA4*	c.2653+23	**G**	C	C	G	G	G	G
chr1:216172380	*USH2A*	c.6506	**A**	A	G	A	A	A	A
chr1:243507682	*SDCCAG8*	c.1473+49	**T**	T	A	T	T	T	T
chr2:29287926	*C2orf71*	c.3676	**C**	C	T	C	C	C	C
chr3:150690476	*CLRN1*	c.20	**T**	A	A	T	T	T	T
chr4:15982166	*PROM1*	c.2374-6	**A**	A	G	A	A	A	A
chr11:61725599	*BEST1*	c.715-19	**A**	C	C	A	A	A	A
chr11:61725615	*BEST1*	c.715-3	**C**	T	T	C	C	C	C
chr11:76895772	*MYO7A*	c.3503+12	**G**	G	A	G	del22	G	del22
NA19258									
chr1:94517166	*ABCA4*	c.2653+23	**G**	C	C	G	G	G	G
chr2:29287926	*C2orf71*	c.3676	**C**	C	T	C	C	C	C
chr3:150690476	*CLRN1*	c.20	**T**	A	A	T	T	T	T
chr4:15982166	*PROM1*	c.2374-6	**A**	A	G	A	A	A	A
chr4:187118042	*CYP4V2*	c.414-52	**C**	T	T	del2	del2	del2	del2
chr11:61725599	*BEST1*	c.715-19	**A**	C	C	A	A	A	A
chr11:61725615	*BEST1*	c.715-3	**C**	T	T	C	C	C	C
chr11:76895772	*MYO7A*	c.3503+12	**G**	G	A	G	del22	G	del22
