# Supplementary material for: Whole Genome Sequencing Increases Molecular Diagnostic Yield Compared with Current Diagnostic Testing for Inherited Retinal Disease
Source: Ophthalmology. 2016 May;123(5):1143–50. doi: 10.1016/j.ophtha.2016.01.009 (PMC4845717; doi:10.1016/j.ophtha.2016.01.009)
Supplement: Table 7 [file mmc6.pdf]

Table 7. Sensitivity of the WGS pipeline for six control samples. Regions of interest include: the hypothesized enrichment region (+/- 50 intronic base pairs of the coding region) for *180 genes* (Table 2, available at http://www.aaojournal.org/); the *exome*, the region classified as protein-coding in the *hg19* reference genome by Complete Genomics; and the *genome*, the whole genomic region. *HG* and *NA* refer to the Coriell Institute for Medical Research (https://www.coriell.org/) identifiers for the DNA samples. Estimated measurements assume a 2.16% error rate in the Illumina OMNI v2.5 microarray.

Coriell ID	# sites surveyed	# observed no-calls	# observed discordances	# observed concordances	True positives	False negatives	Sensitivity	Estimated False negatives	Estimated Sensitivity
**180-genes**									
HG01970	3676	23	134	3519	3519	157	0.957	78	0.978
NA18533	3937	23	156	3758	3758	179	0.955	94	0.976
NA18907	4434	26	138	4270	4270	164	0.963	68	0.984
NA19005	4011	27	173	3811	3811	200	0.950	113	0.971
NA19194	4436	30	145	4258	4258	178	0.960	82	0.981
NA19258	4418	38	148	4232	4232	186	0.958	91	0.979
							**0.957**		**0.978**
**exome**									
HG01970	20030	142	499	19389	19389	641	0.968	208	0.989
NA18533	21218	130	543	20545	20545	673	0.968	215	0.990
NA18907	24909	160	491	24258	24258	651	0.974	113	0.995
NA19005	20844	174	629	20041	20041	803	0.961	353	0.983
NA19194	24453	212	591	23650	23650	803	0.967	275	0.989
NA19258	24181	273	555	23353	23353	828	0.966	306	0.987
							**0.967**		**0.989**
**genome**									
HG01970	701532	6403	24237	670892	670892	30640	0.956	15487	0.977
NA18533	734586	6850	26081	701655	701655	32931	0.955	17064	0.976
NA18907	826732	8754	24514	793464	793464	33268	0.960	15411	0.981
NA19005	731713	7117	29080	695516	695516	36197	0.951	20392	0.972
NA19194	804975	8859	27170	768945	768945	36029	0.955	18642	0.976
NA19258	805704	8278	26146	771279	771279	34424	0.957	17021	0.978
							**0.956**		**0.977**
