# Supplementary material for: Whole Genome Sequencing Increases Molecular Diagnostic Yield Compared with Current Diagnostic Testing for Inherited Retinal Disease
Source: Ophthalmology. 2016 May;123(5):1143–50. doi: 10.1016/j.ophtha.2016.01.009 (PMC4845717; doi:10.1016/j.ophtha.2016.01.009)
Supplement: Table 10 [file mmc8.pdf]

Table 10. Variants accounting for clinical diagnosis in the 46 patients referred for WGS. *Red text* indicates variants previously identified through targeted NGS testing. Confirmations of variants identified exclusively through the WGS pipeline (*black text*) are available at http://www.aaojournal.org/ (Figures 4-17). Annotations were performed on the transcripts included in Table 1 (available at http://www.aaojournal.org/).

Study ID	Gene	Allele 1	Allele 2	Suspected disease inheritance	Referral patient group
12003183	***CRX***	c.648delC^	-	ad	A_2
065240	***MERTK***	c.-8163_c.1145-1213del[1]	c.-8163_c.1145-1213del[1]	ar	A_2
11012959	***TRPM1***	c.707T>C p.(Leu236Pro)^	c.707T>C p.(Leu236Pro)^	ar	A_2
12007903	***ABCA4***	c.2041C>T p.(Arg681Ter)[2,3]	c.5461-10T>C[4-8]	ar	A_1
065238	***ABCA4***	c.4577C>T p.(Thr1526Met)[3,9,10]	c.5714+5G>A[7,11,12]	ar	A_1
09006916	***ABCA4***	c.1335C>G p.(Ser445Arg)[13,14]	c.5461-10T>C[4-8]	ar	A_1
11001193	***PDE6B***	c.1876G>T p.(Glu626Ter)^	c.1923_1969delinsTCTGGG p.(Asn643Glyfs*29)^	ar	A_1
11012351	***GPR98***	c.1239-8C>G^	c.16079-1455_c.16196+155del^	ar	A_1
11013807	***USH2A***	c.4474G>T p.(Glu1492Ter)[15-17]	c.5614delinsTTAACTTGGCAT^	ar	A_1
12002355	***PCDH15***	c.2986C>T p.(Arg966Ter)[18-20]	c.-189197_c.610-5166del^	ar	A_1
13012708	***ABCA4***	c.4537dupC[21]	c.5714+5G>A[7,11,12]	ar	A_1
10002008	***MYO7A***	c.6025delG[22,23]	c.4115T>G p.(Val1372Gly)^	ar	B_2
084928	***RBP3***	c.249C>A p.(Asn83Lys)^	c.1237C>T p.(Pro413Ser)^	ar	B_2
12008422	***USH2A***	c.6446C>A p.(Pro2149Gln)^	c.6326-3582_6658-1028del^	ar	B_2
09001814	***RS1***	c.304C>T p.(Arg102Trp)[24-26]	-	xl	B_1
087384	***TOPORS***	c.2539C>T p.(Arg847Ter)^	-	ad	B_1
083869	***ABCA4***	c.2713delG^	c.2713delG^	ar	B_1
10009624	***PROM1***	c.1726C>T p.(Gln576Ter)[27]	c.1726C>T p.(Gln576Ter)[27]	ar	B_1
070553	***RPGR***	c.1928C>G p.(Ser643Ter)[28]	-	xl	B_1
087001	***USH2A***	c.2299delG[17,29]	c.4321G>T p.(Glu1441Ter)^	ar	B_1
09000190	***ABCA4***	c.4918C>T p.(Arg1640Trp)[30]	c.4469G>A p.(Cys1490Tyr)[31]	ar/complex	B_1
		c.4222T>C p.(Trp1408Arg)[30]			
067099	***PRPF31***	c.1120C>T p.(Gln374Ter)^		ad	B_1
067429	***EYS***	c.7095T>G p.(Tyr2365Ter)[32]	c.9277_9278dupGG^	ar	B_1
087751	***BBS1***	c.1169T>G p.(Met390Arg)[33,34]	c.1169T>G p.(Met390Arg)[33,34]	ar	B_1

^ a 'likely pathogenic' variant which had not been previously reported in the literature at the time of clinical analysis.

1. Ostergaard, E., Duno, M., Batbayli, M., Vilhelmsen, K. & Rosenberg, T. A novel MERTK deletion is a common founder mutation in the Faroe Islands and is responsible for a high proportion of retinitis pigmentosa cases. *Mol Vis* **17**, 1485-92 (2011).
2. Maugeri, A. et al. Mutations in the ABCA4 (ABCR) gene are the major cause of autosomal recessive cone-rod dystrophy. *Am J Hum Genet* **67**, 960-6 (2000).
3. Cideciyan, A.V. et al. ABCA4 disease progression and a proposed strategy for gene therapy. *Hum Mol Genet* **18**, 931-41 (2009).
4. Jonsson, F., Burstedt, M.S., Sandgren, O., Norberg, A. & Golovleva, I. Novel mutations in CRB1 and ABCA4 genes cause Leber congenital amaurosis and Stargardt disease in a Swedish family. *Eur J Hum Genet* **21**, 1266-71 (2013).
5. Zernant, J. et al. Analysis of the ABCA4 gene by next-generation sequencing. *Invest Ophthalmol Vis Sci* **52**, 8479-87 (2011).
6. Fujinami, K. et al. ABCA4 gene screening by next-generation sequencing in a British cohort. *Invest Ophthalmol Vis Sci* **54**, 6662-74 (2013).
7. Kitiratschky, V.B.D. et al. ABCA4 gene analysis in patients with autosomal recessive cone and cone rod dystrophies. *European Journal of Human Genetics* **16**, 812-819 (2008).
8. Utz, V.M. et al. Predictors of visual acuity and genotype-phenotype correlates in a cohort of patients with Stargardt disease. *British Journal of Ophthalmology* **98**, 513-518 (2014).
9. Lewis, R.A. et al. Genotype/Phenotype analysis of a photoreceptor-specific ATP-binding cassette transporter gene, ABCR, in Stargardt disease. *Am J Hum Genet* **64**, 422-34 (1999).
10. Sun, H., Smallwood, P.M. & Nathans, J. Biochemical defects in ABCR protein variants associated with human retinopathies. *Nat Genet* **26**, 242-6 (2000).
11. Cremers, F.P.M. et al. Autosomal recessive retinitis pigmentosa and cone-rod dystrophy caused by splice site mutations in the Stargardt's disease gene ABCR. *Human Molecular Genetics* **7**, 355-362 (1998).
12. Klevering, B.J. et al. Phenotypic variations in a family with retinal dystrophy as result of different mutations in the ABCR gene. *Br J Ophthalmol* **83**, 914-8 (1999).
13. Papaioannou, M. et al. An analysis of ABCR mutations in British patients with recessive retinal dystrophies. *Invest Ophthalmol Vis Sci* **41**, 16-9 (2000).
14. Webster, A.R. et al. An analysis of allelic variation in the ABCA4 gene. *Invest Ophthalmol Vis Sci* **42**, 1179-89 (2001).
15. Garcia-Garcia, G. et al. Mutational screening of the USH2A gene in Spanish USH patients reveals 23 novel pathogenic mutations. *Orphanet J Rare Dis* **6**, 65 (2011).
16. Bernal, S. et al. Clinical and genetic studies in Spanish patients with Usher syndrome type II: description of new mutations and evidence for a lack of genotype–phenotype correlation. *Clinical Genetics* **68**, 204-214 (2005).
17. Le Quesne Stabej, P. et al. Comprehensive sequence analysis of nine Usher syndrome genes in the UK National Collaborative Usher Study. *J Med Genet* **49**, 27-36 (2012).
18. Roux, A.F. et al. Survey of the frequency of USH1 gene mutations in a cohort of Usher patients shows the importance of cadherin 23 and protocadherin 15 genes and establishes a detection rate of above 90%. *Journal of Medical Genetics* **43**, 763-768 (2006).
19. Xiong, H.Y. et al. The human splicing code reveals new insights into the genetic determinants of disease. *Science* **347**, 144-+ (2015).
20. Yoshimura, H. et al. Massively parallel DNA sequencing facilitates diagnosis of patients with Usher syndrome type 1. *Plos One* **9**, e90688 (2014).

21. Briggs, C.E. et al. Mutations in ABCR (ABCA4) in patients with Stargardt macular degeneration or cone-rod degeneration. *Investigative Ophthalmology & Visual Science* **42**, 2229-2236 (2001).
22. Gerber, S. et al. USH1A: chronicle of a slow death. *Am J Hum Genet* **78**, 357-9 (2006).
23. Roux, A.F. et al. Four-year follow-up of diagnostic service in USH1 patients. *Invest Ophthalmol Vis Sci* **52**, 4063-71 (2011).
24. Sauer, C.G. et al. Positional cloning of the gene associated with X-linked juvenile retinoschisis. *Nat Genet* **17**, 164-70 (1997).
25. Wang, T. et al. Intracellular retention of mutant retinoschisin is the pathological mechanism underlying X-linked retinoschisis. *Hum Mol Genet* **11**, 3097-105 (2002).
26. Vijayasarathy, C. et al. Molecular mechanisms leading to null-protein product from retinoschisin (RS1) signal-sequence mutants in X-linked retinoschisis (XLRS) disease. *Hum Mutat* **31**, 1251-60 (2010).
27. Zhang, Q. et al. Severe retinitis pigmentosa mapped to 4p15 and associated with a novel mutation in the PROM1 gene. *Hum Genet* **122**, 293-9 (2007).
28. Shu, X. et al. RPGR mutation analysis and disease: an update. *Hum Mutat* **28**, 322-8 (2007).
29. Eudy, J.D. et al. Mutation of a gene encoding a protein with extracellular matrix motifs in Usher syndrome type IIa. *Science* **280**, 1753-7 (1998).
30. Shroyer, N.F., Lewis, R.A., Yatsenko, A.N., Wensel, T.G. & Lupski, J.R. Cosegregation and functional analysis of mutant ABCR (ABCA4) alleles in families that manifest both Stargardt disease and age-related macular degeneration. *Hum Mol Genet* **10**, 2671-8 (2001).
31. Wiszniewski, W. et al. ABCA4 mutations causing mislocalization are found frequently in patients with severe retinal dystrophies. *Hum Mol Genet* **14**, 2769-78 (2005).
32. Littink, K.W. et al. Mutations in the EYS gene account for approximately 5% of autosomal recessive retinitis pigmentosa and cause a fairly homogeneous phenotype. *Ophthalmology* **117**, 2026-33, 2033 e1-7 (2010).
33. Davis, R.E. et al. A knockin mouse model of the Bardet-Biedl syndrome 1 M390R mutation has cilia defects, ventriculomegaly, retinopathy, and obesity. *Proc Natl Acad Sci U S A* **104**, 19422-7 (2007).
34. Beales, P.L. et al. Genetic interaction of BBS1 mutations with alleles at other BBS loci can result in non-Mendelian Bardet-Biedl syndrome. *Am J Hum Genet* **72**, 1187-99 (2003).
