# Supplementary material for: Whole Genome Sequencing Increases Molecular Diagnostic Yield Compared with Current Diagnostic Testing for Inherited Retinal Disease
Source: Ophthalmology. 2016 May;123(5):1143–50. doi: 10.1016/j.ophtha.2016.01.009 (PMC4845717; doi:10.1016/j.ophtha.2016.01.009)
Supplement: Table 11 [file mmc9.pdf]

Table 11. Intronic variants outside the +5/-5 intronic regions of coding transcripts now surveyed by targeted NGS.

Gene	cDNA	Genomic co-ordinate
ABCA4	c.4539+2001 G>A	chr1:94493000
ABCA4	c.4539+2028 C>T	chr1:94492973
ABCA4	c.5196+1056 A>G	chr1:94484082
ABCA4	c.5196+1137 G>A	chr1:94484001
ABCA4	c.5196+1216 C>A	chr1:94483922
ABCA4	c.5461-10 T>C	chr1:94476951
CEP290	c.2991+1655 A>G	chr12:88494960
OFD1	c.935+706 A>G	chrX:13768358
USH2A	c.7595-2144 A>G	chr1:216064540
