# Supplementary material for: Whole Genome Sequencing Increases Molecular Diagnostic Yield Compared with Current Diagnostic Testing for Inherited Retinal Disease
Source: Ophthalmology. 2016 May;123(5):1143–50. doi: 10.1016/j.ophtha.2016.01.009 (PMC4845717; doi:10.1016/j.ophtha.2016.01.009)
Supplement: Figure 4 [file mmc10.pdf]

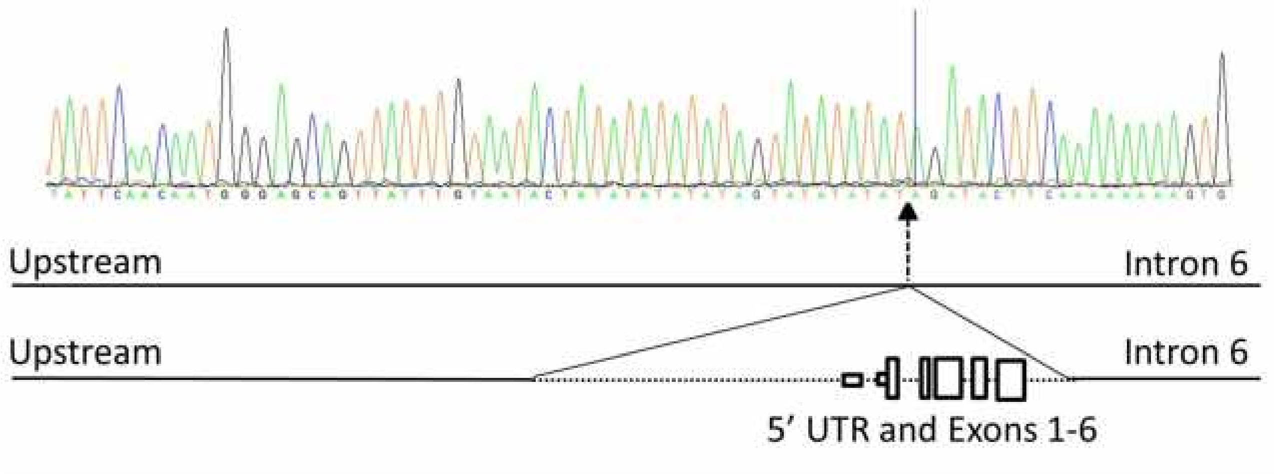

Figure 4. Sanger sequencing chromatogram for patient 12002355, showing a heterozygous deletion removing the start codon and first 6 coding exons of the *PCDH15* gene (chr10:56,094,632-56,613,219; *PCDH15* c.-189197_c.610-5166, NM_001142763.1). Large structural rearrangements have previously been assigned as the cause of Usher syndrome in the *PCDH15* gene, including single and multiple exon deletions.[1-3]

1. Jaijo T, Oshima A, Aller E, et al. Mutation screening of the PCDH15 gene in Spanish patients with Usher syndrome type I. Molecular Vision 2012;18:1719-26.
2. Roux AF, Faugere V, Le Guedard S, et al. Survey of the frequency of USH1 gene mutations in a cohort of Usher patients shows the importance of cadherin 23 and protocadherin 15 genes and establishes a detection rate of above 90%. Journal of Medical Genetics 2006;43:763-8.
3. Glockle N, Kohl S, Mohr J, et al. Panel-based next generation sequencing as a reliable and efficient technique to detect mutations in unselected patients with retinal dystrophies. Eur J Hum Genet 2014;22:99-104.
