# Supplementary material for: Whole Genome Sequencing Increases Molecular Diagnostic Yield Compared with Current Diagnostic Testing for Inherited Retinal Disease
Source: Ophthalmology. 2016 May;123(5):1143–50. doi: 10.1016/j.ophtha.2016.01.009 (PMC4845717; doi:10.1016/j.ophtha.2016.01.009)
Supplement: Figure 5 [file mmc11.pdf]

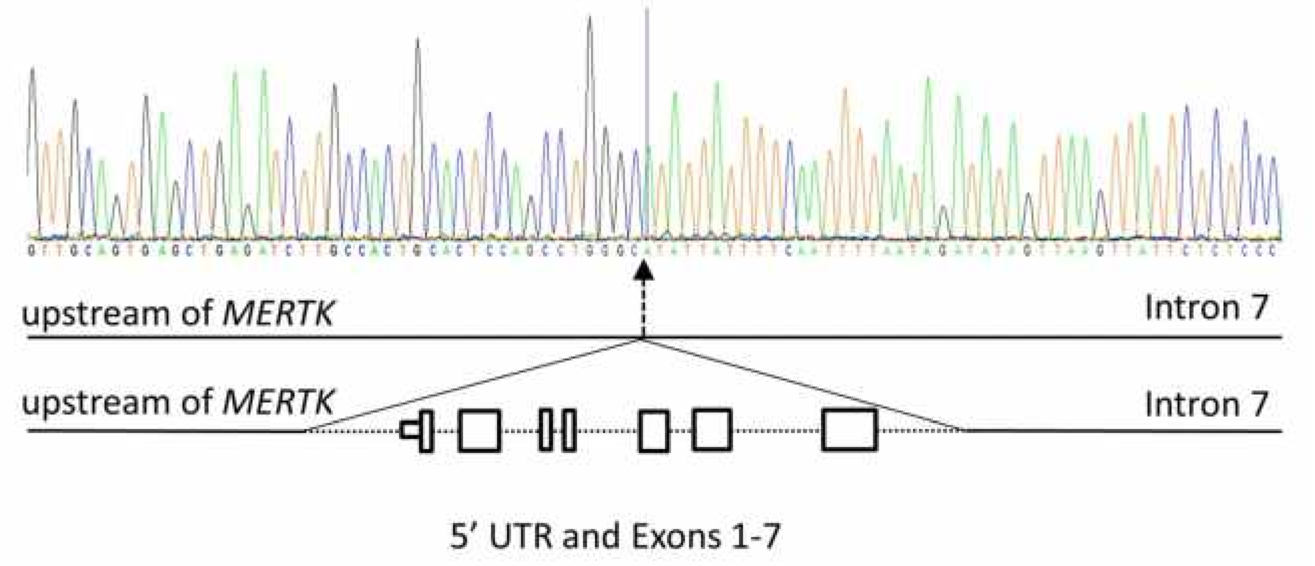

Figure 5. Sanger sequencing chromatogram for patient 065240, showing a homozygous deletion removing the start codon and first 7 coding exons of the *MERTK* gene (chr2:112,648,150-112,739,206; *MERTK* c.-8163_c.1145-1213, NM_006343.2). c.-8163_c.1145-1213 has not been reported in the literature previously, but an almost identical 91kb deletion which also removes the first 7 coding exons, has been reported as in a homozygous state in patients with retinitis pigmentosa.[1]

1. Ostergaard E, Duno M, Batbayli M, Vilhelmsen K, Rosenberg T. A novel MERTK deletion is a common founder mutation in the Faroe Islands and is responsible for a high proportion of retinitis pigmentosa cases. Mol Vis 2011;17:1485-92.
