# Supplementary material for: Whole Genome Sequencing Increases Molecular Diagnostic Yield Compared with Current Diagnostic Testing for Inherited Retinal Disease
Source: Ophthalmology. 2016 May;123(5):1143–50. doi: 10.1016/j.ophtha.2016.01.009 (PMC4845717; doi:10.1016/j.ophtha.2016.01.009)
Supplement: Figure 6 [file mmc12.pdf]

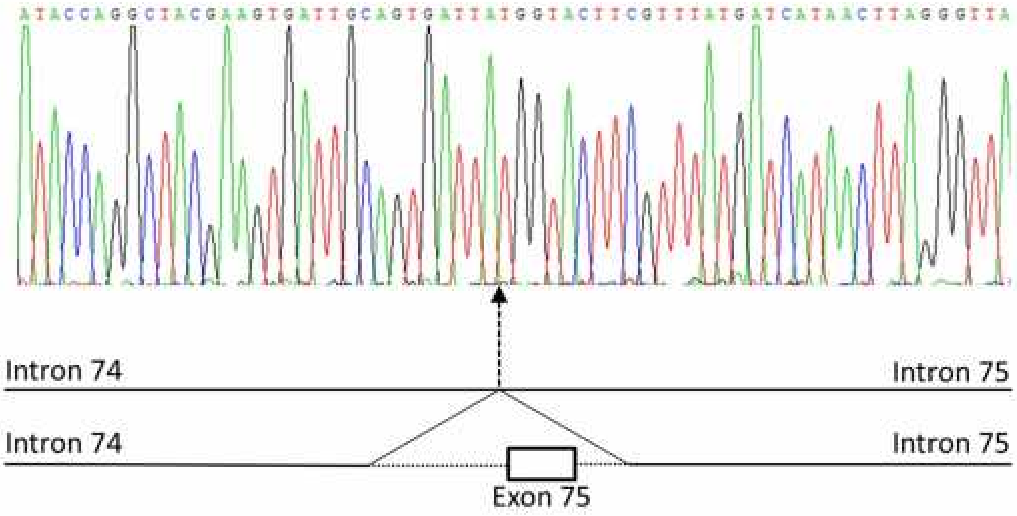

Figure 6. Sanger sequencing chromatogram for patient 11012351, showing a heterozygous deletion removing a single exon from the reading frame of the *GPR98* gene (chr5:90,109,981-90,111,708; *GPR98* c.16079-1455_c.16196+155del, p.(Ser5361Profs*25); NM_032119.3). c.16079-1455_ c.16196+155del has not been previously reported in the literature, however it is expected to cause the deletion of exon 75 and flanking intronic regions of *GPR98*. Deletions that cause the removal of whole exons in GPR98 have previously been reported in patients with Usher syndrome,[1] and the removal of exon 75 is predicted to cause premature termination of the *GPR98* protein product.

1. Hilgert N, Kahrizi K, Dieltjens N, et al. A large deletion in GPR98 causes type IIC Usher syndrome in male and female members of an Iranian family. J Med Genet 2009;46:272-6.
