# Supplementary material for: Whole Genome Sequencing Increases Molecular Diagnostic Yield Compared with Current Diagnostic Testing for Inherited Retinal Disease
Source: Ophthalmology. 2016 May;123(5):1143–50. doi: 10.1016/j.ophtha.2016.01.009 (PMC4845717; doi:10.1016/j.ophtha.2016.01.009)
Supplement: Figure 7 [file mmc13.pdf]

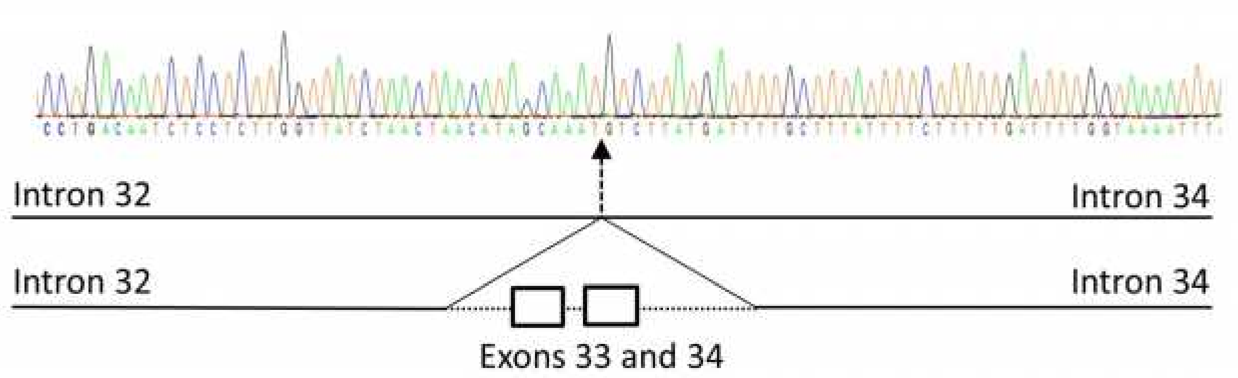

Figure 7. Sanger sequencing chromatogram for patient 12008422, showing a heterozygous deletion removing two exons from the reading frame from the *USH2A* gene (chr1:216,167,537-216,177,486; *USH2A* c.6326-3582_6658-1028del, p.(Asp2109Glyfs*11), NM_206933.2). Heterozygous whole exon deletions have previously been reported as pathogenic in the *USH2A* gene.[1,2]

1. Baux D, Blanchet C, Hamel C, et al. Enrichment of LOVD-USHbases with 152 USH2A Genotypes Defines an Extensive Mutational Spectrum and Highlights Missense Hotspots. Hum Mutat 2014.
2. Krawitz PM, Schiska D, Krüger U, et al. Screening for single nucleotide variants, small indels and exon deletions with a next-generation sequencing based gene panel approach for Usher syndrome. Molecular Genetics & Genomic Medicine 2014:n/a-n/a.
