# Supplementary material for: Whole Genome Sequencing Increases Molecular Diagnostic Yield Compared with Current Diagnostic Testing for Inherited Retinal Disease
Source: Ophthalmology. 2016 May;123(5):1143–50. doi: 10.1016/j.ophtha.2016.01.009 (PMC4845717; doi:10.1016/j.ophtha.2016.01.009)
Supplement: Figure 8 [file mmc14.pdf]

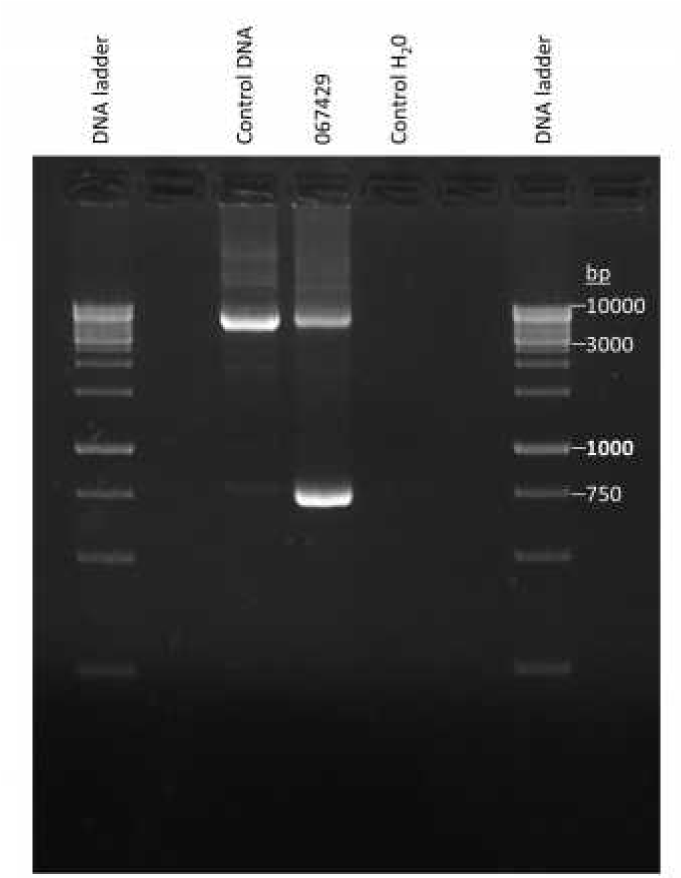

Figure 8. Gel electrophoresis of PCR products for patient 067429, showing a heterozygous deletion removing three exons from the reading frame of the *RPGRIP1* gene (chr14:21,794,817-21,799,356; *RPGRIP1* c.2710+485_3238+810del, p.(Gly904_Asn1079del), NM_020366.3). c.2710+485_3238+810del has not been reported previously, but is expected to remove three exons from the reading frame and 175 amino acids from the protein primary structure. The deletion keeps an inframe reading frame and is not expected to cause a premature termination of protein product. Homozygous and compound heterozygous mutations in RPGRIP1 have previously been reported as a cause of Leber congenital amaurosis[1] and cone-rod dystrophy.[2] No other clearly pathogenic variant is found in *RPGRIP1* in this patient's DNA. Therefore, c.2710+485_3238+810del is interpreted as potentially pathogenic but not disease-causing.

1. Dryja TP, Adams SM, Grimsby JL, et al. Null RPGRIP1 alleles in patients with Leber congenital amaurosis. Am J Hum Genet 2001;68:1295-8.
2. Hameed A, Abid A, Aziz A, Ismail M, Mehdi SQ, Khaliq S. Evidence of RPGRIP1 gene mutations associated with recessive cone-rod dystrophy. J Med Genet 2003;40:616-9.
