# Supplementary material for: Whole Genome Sequencing Increases Molecular Diagnostic Yield Compared with Current Diagnostic Testing for Inherited Retinal Disease
Source: Ophthalmology. 2016 May;123(5):1143–50. doi: 10.1016/j.ophtha.2016.01.009 (PMC4845717; doi:10.1016/j.ophtha.2016.01.009)
Supplement: Figure 10 [file mmc16.pdf]

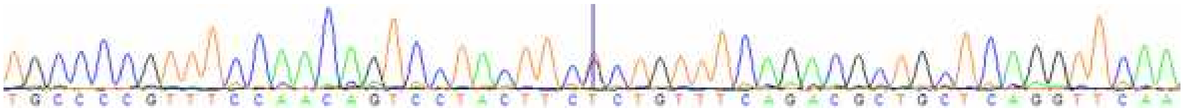

Figure 10. Sanger sequencing chromatogram for patient 12007903, showing a heterozygous intronic variant in the *ABCA4* gene (chr1:94,476,951; *ABCA4* c.5461-10T>C, NM_000350.2). c.5461-10T>C is known to segregate with clinical presentation of IRD,[1-4] but functional mechanism of pathogenesis is unknown.

1. Jonsson F, Burstedt MS, Sandgren O, Norberg A, Golovleva I. Novel mutations in CRB1 and ABCA4 genes cause Leber congenital amaurosis and Stargardt disease in a Swedish family. Eur J Hum Genet 2013;21:1266-71.
2. Zernant J, Schubert C, Im KM, et al. Analysis of the ABCA4 gene by next-generation sequencing. Invest Ophthalmol Vis Sci 2011;52:8479-87.
3. Fujinami K, Zernant J, Chana RK, et al. ABCA4 gene screening by next-generation sequencing in a British cohort. Invest Ophthalmol Vis Sci 2013;54:6662-74.
4. Utz VM, Coussa RG, Marino MJ, et al. Predictors of visual acuity and genotype-phenotype correlates in a cohort of patients with Stargardt disease. Br J Ophthalmol 2014;98:513-8.
