# Supplementary material for: Whole Genome Sequencing Increases Molecular Diagnostic Yield Compared with Current Diagnostic Testing for Inherited Retinal Disease
Source: Ophthalmology. 2016 May;123(5):1143–50. doi: 10.1016/j.ophtha.2016.01.009 (PMC4845717; doi:10.1016/j.ophtha.2016.01.009)
Supplement: Figure 11 [file mmc17.pdf]

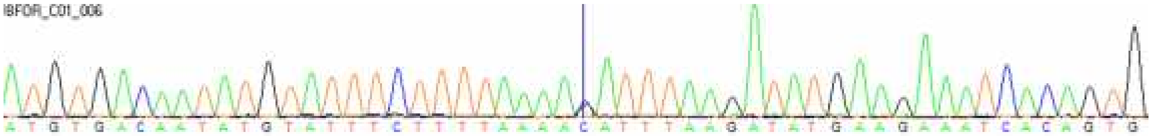

Figure 11. Sanger sequencing chromatogram for patient 11012351, showing a heterozygous intronic variant predicted to affect splicing in the *GPR98* gene (chr5:89,924,371; *GPR98* c.1239-8C>G, NM_032119.3). c.1239-8C>G has not previously been reported in the literature, however *in-silico* predictions suggest that it introduces a strong 3' splicing site before the 3' canonical splicing site of exon 8. Mutations that disrupt splicing in *GPR98* have been previously reported as pathogenic in patients with Usher syndrome.[1,2]

1. Le Quesne Stabej P, Saihan Z, Rangesh N, et al. Comprehensive sequence analysis of nine Usher syndrome genes in the UK National Collaborative Usher Study. J Med Genet 2012;49:27-36.
2. Garcia-Garcia G, Besnard T, Baux D, et al. The contribution of GPR98 and DFNB31 genes to a Spanish Usher syndrome type 2 cohort. Mol Vis 2013;19:367-73.
