# Supplementary material for: Whole Genome Sequencing Increases Molecular Diagnostic Yield Compared with Current Diagnostic Testing for Inherited Retinal Disease
Source: Ophthalmology. 2016 May;123(5):1143–50. doi: 10.1016/j.ophtha.2016.01.009 (PMC4845717; doi:10.1016/j.ophtha.2016.01.009)
Supplement: Figure 12 [file mmc18.pdf]

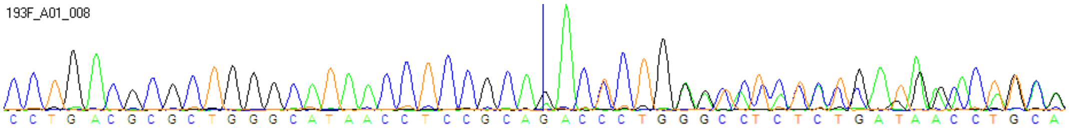

Figure 12. Sanger sequencing chromatogram for patient 11001193, showing a heterozygous deletion which removes part of the coding sequence of the *PDE6B* gene (chr4:657,561-657,607; *PDE6B* c.1923_1969delinsTCTGGG, p.(Asn643Glyfs*29), NM_000283.3). c.1923_1969delinsTCTGGG is predicted to disrupt the normal translation of the *PDE6B* gene and a different frameshift mutation, c.1927_1969delinsGG, which has the same predicted protein change, p.(Asn643Glyfs*29), has been reported previously in a compound heterozygous state in patients with retinitis pigmentosa.[1]

1. Neveling K, Collin RWJ, Gilissen C, et al. Next-Generation Genetic Testing for Retinitis Pigmentosa. Human Mutation 2012;33:963-72.
