# Supplementary material for: Whole Genome Sequencing Increases Molecular Diagnostic Yield Compared with Current Diagnostic Testing for Inherited Retinal Disease
Source: Ophthalmology. 2016 May;123(5):1143–50. doi: 10.1016/j.ophtha.2016.01.009 (PMC4845717; doi:10.1016/j.ophtha.2016.01.009)
Supplement: Figure 13 [file mmc19.pdf]

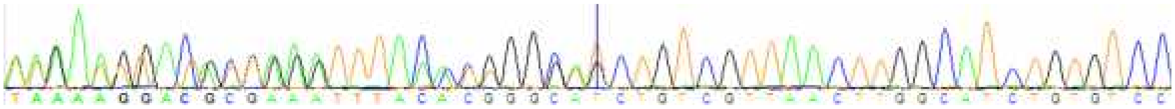

Figure 13. Sanger sequencing chromatogram for patient 11013807, showing a heterozygous insertion-deletion which removes part of the coding sequence of the *USH2A* gene (chr1:216,246,601; *USH2A* c.5614delinsTTAACTTGGCAT, p.(Ala1872Metfs*4), NM_206933.2). c.5614delinsTTAACTTGGCAT is expected to cause a shift in the reading frame and a premature termination of the USH2A protein product. An indel event at the same protein position as the one reported here, p.Ala1872Leufs*58, has been previously reported in a compound heterozygous state in patients with Usher Syndrome Type II.[1]

1. Le Quesne Stabej P, Saihan Z, Rangesh N, et al. Comprehensive sequence analysis of nine Usher syndrome genes in the UK National Collaborative Usher Study. J Med Genet 2012;49:27-36.
