# Supplementary material for: Whole Genome Sequencing Increases Molecular Diagnostic Yield Compared with Current Diagnostic Testing for Inherited Retinal Disease
Source: Ophthalmology. 2016 May;123(5):1143–50. doi: 10.1016/j.ophtha.2016.01.009 (PMC4845717; doi:10.1016/j.ophtha.2016.01.009)
Supplement: Figure 14 [file mmc20.pdf]

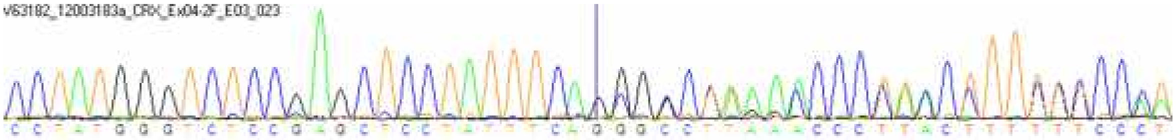

Figure 14. Sanger sequencing chromatogram for patient 12003183, showing a heterozygous single nucleotide deletion in the *CRX* gene (chr19:48342972; *CRX* c.648delC, p.(Ser216Argfs*3), NM_000554.4). c.648delC has not been reported previously in patients with retinitis pigmentosa, however it is predicted to terminate the CRX protein prematurely. Truncating mutations in CRX have been previously reported in patients with autosomal dominant Leber congenital amaurosis.[1-3]

1. Freund CL, Wang QL, Chen S, et al. De novo mutations in the CRX homeobox gene associated with Leber congenital amaurosis. Nat Genet 1998;18:311-2.
2. Sohocki MM, Sullivan LS, Mintz-Hittner HA, et al. A range of clinical phenotypes associated with mutations in CRX, a photoreceptor transcription-factor gene. Am J Hum Genet 1998;63:1307-15.
3. Arcot Sadagopan K, Battista R, Keep RB, Capasso JE, Levin AV. Autosomal-dominant Leber Congenital Amaurosis Caused by a Heterozygous CRX Mutation in a Father and Son. Ophthalmic Genet 2013.
