# Supplementary material for: Whole Genome Sequencing Increases Molecular Diagnostic Yield Compared with Current Diagnostic Testing for Inherited Retinal Disease
Source: Ophthalmology. 2016 May;123(5):1143–50. doi: 10.1016/j.ophtha.2016.01.009 (PMC4845717; doi:10.1016/j.ophtha.2016.01.009)
Supplement: Figure 15 [file mmc21.pdf]

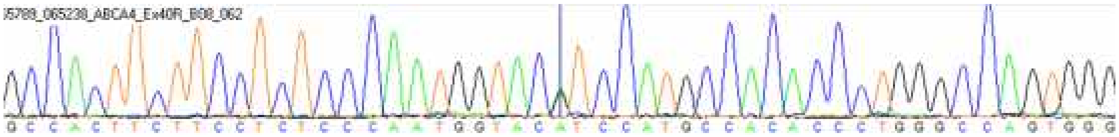

Figure 15. Sanger sequencing chromatogram for patient 065238, showing a heterozygous intronic variant in the *ABCA4* gene (chr1:94,476,351; *ABCA4* c.5714+5G>A, NM_000350.2). c.5714+5G>A reduces the splicing efficiency of the exon40/intron40 donor splice site[1] and has previously been reported as pathogenic.[2,3]

1. Rivera A, White K, Stohr H, et al. A comprehensive survey of sequence variation in the ABCA4 (ABCR) gene in Stargardt disease and age-related macular degeneration. Am J Hum Genet 2000;67:800-13.
2. Cremers FPM, van De Pol DJR, van Driel M, et al. Autosomal recessive retinitis pigmentosa and cone-rod dystrophy caused by splice site mutations in the Stargardt's disease gene ABCR. Human Molecular Genetics 1998;7:355-62.
3. Klevering BJ, van Driel M, van de Pol DJ, Pinckers AJ, Cremers FP, Hoyng CB. Phenotypic variations in a family with retinal dystrophy as result of different mutations in the ABCR gene. Br J Ophthalmol 1999;83:914-8.
