# Supplementary material for: Whole Genome Sequencing Increases Molecular Diagnostic Yield Compared with Current Diagnostic Testing for Inherited Retinal Disease
Source: Ophthalmology. 2016 May;123(5):1143–50. doi: 10.1016/j.ophtha.2016.01.009 (PMC4845717; doi:10.1016/j.ophtha.2016.01.009)
Supplement: Table 8 [file mmc7.pdf]

Table 8. Specificity of the WGS pipeline for six control samples. Regions of interest include: the clinically analysed region of *105 genes* (Table 1, available at http://www.aaojournal.org/); the hypothesized enrichment region (+/- 50 intronic base pairs of the coding region) for *180 genes* (Table 2, available at http://www.aaojournal.org/); the *exome*, the region classified as protein-coding in the *hg19* reference genome by Complete Genomics; and the *genome*, the whole genomic region. *HG* and *NA* refer to the Coriell Institute for Medical Research (https://www.coriell.org/) identifiers for the DNA samples.

Coriell ID	# sites surveyed	# observed no-calls	# observed discordances	# observed concordances	True negatives	False positives	Specificity
**105-genes**							
HG01970	568	0	0	568	568	0	1.0
NA18533	554	0	0	554	554	0	1.0
NA18907	519	1	0	518	518	1	0.998
NA19005	554	0	0	554	554	0	1.0
NA19194	523	0	1	522	522	1	0.998
NA19258	520	0	0	520	520	0	1.0
							**0.999**
**180-genes**							
HG01970	10144	27	27	10090	10090	71	0.993
NA18533	9575	31	28	9516	9516	59	0.994
NA18907	9348	38	29	9281	9281	67	0.993
NA19005	9609	37	34	9538	9538	71	0.993
NA19194	9318	48	35	9235	9235	83	0.991
NA19258	9346	41	34	9271	9271	75	0.992
							**0.993**
**exome**							
HG01970	71071	189	137	70745	67833	317	0.996
NA18533	66026	236	140	65650	65650	376	0.994
NA18907	66023	171	131	65721	65721	302	0.995
NA19005	68150	199	118	67833	67833	317	0.995
NA19194	65793	197	126	65470	65470	323	0.995
NA19258	68717	154	119	68444	68444	273	0.996
							**0.995**
**genome**							
HG01970	1738575	10829	6646	1721100	1721100	17715	0.990
NA18533	1664874	11354	6393	1647127	1647127	17747	0.989
NA18907	1605493	12513	6600	1586380	1586380	19113	0.988
NA19005	1676548	11131	6584	1658833	1658833	17715	0.989
NA19194	1621888	12911	6779	1602198	1602198	19690	0.988
NA19258	1621311	11714	6717	1602880	1602880	18431	0.989
							**0.989**
