# Supplementary material for: Whole Genome Sequencing Increases Molecular Diagnostic Yield Compared with Current Diagnostic Testing for Inherited Retinal Disease
Source: Ophthalmology. 2016 May;123(5):1143–50. doi: 10.1016/j.ophtha.2016.01.009 (PMC4845717; doi:10.1016/j.ophtha.2016.01.009)
Supplement: Appendix [file mmc24.pdf]

Case Study – Usher syndrome

A female presented with retinitis pigmentosa (RP) and congenital deafness suggesting a clinical diagnosis of Usher syndrome. There was no family history.

Targeted NGS testing identified no molecular cause. The patient carried:

i) a ‘likely pathogenic’ heterozygous variant in *IDH3B*, c.184G>T, p.(Glu62*).

Mutations in *IDH3B* have previously been associated with autosomal recessive non-syndromic RP.[30] This finding, therefore, was concluded not to contribute to the molecular diagnosis of IRD for this individual.

Analysis of WGS data additionally identified:

ii) a ‘likely pathogenic’ heterozygous *GPR98* non-coding variant, c.1239-8C>G

iii) a ‘likely pathogenic’ heterozygous deletion, c.16079-1456_ c.16196+155del p.(Ser5361Profs*25), removing a protein-coding region (exon) of *GPR98* (Figure 3) and expected to cause premature termination of protein translation.

Homozygous and compound heterozygous mutations in *GPR98* cause Usher syndrome.[2] These findings were therefore concluded to confirm a ***diagnosis of autosomal recessive Usher syndrome.***
