# Supplementary material for: Whole Genome Sequencing Increases Molecular Diagnostic Yield Compared with Current Diagnostic Testing for Inherited Retinal Disease
Source: Ophthalmology. 2016 May;123(5):1143–50. doi: 10.1016/j.ophtha.2016.01.009 (PMC4845717; doi:10.1016/j.ophtha.2016.01.009)
Supplement: Figure 17 [file mmc23.pdf]

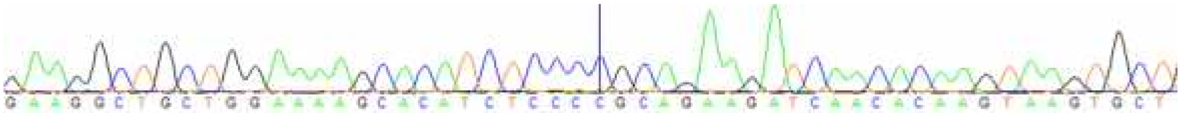

Figure 17. Sanger sequencing chromatogram for patient 11012959, showing a homozygous missense variant in the *TRPM1* gene (chr15:31,358,296; *TRPM1* c.707T>C, p.(Leu236Pro), NM_002420.5). c.707T>C has not previously been reported in patients with recessively inherited congenital stationary night blindness (CSNB), nor has it been reported as a normal polymorphism. However, missense mutations in *TRPM1* have previously been reported in a recessive state in patients with CSNB,[1-3] and *in-silico* analysis suggests that c.707T>C will have a detrimental effect on the TRPM1 protein.

1. van Genderen MM, Bijveld MM, Claassen YB, et al. Mutations in TRPM1 are a common cause of complete congenital stationary night blindness. Am J Hum Genet 2009;85:730-6.
2. Sergouniotis PI, Chakarova C, Murphy C, et al. Biallelic variants in TTLL5, encoding a tubulin glutamylase, cause retinal dystrophy. Am J Hum Genet 2014;94:760-9.
3. Audo I, Kohl S, Leroy BP, et al. TRPM1 is mutated in patients with autosomal-recessive complete congenital stationary night blindness. Am J Hum Genet 2009;85:720-9.
